# Solvothermal Preparation of a Lanthanide Metal-Organic Framework for Highly Sensitive Discrimination of Nitrofurantoin and l-Tyrosine

**DOI:** 10.3390/molecules26123673

**Published:** 2021-06-16

**Authors:** Tian-Tian Wang, Jing-Yi Liu, Rui Guo, Jun-Dan An, Jian-Zhong Huo, Yuan-Yuan Liu, Wei Shi, Bin Ding

**Affiliations:** 1 Key Laboratory of Inorganic-Organic Hybrid Functional Material Chemistry, College of Chemistry, Tianjin Normal University, 393 Binshui West Road, Tianjin 300387, China; tiantianwang0912@163.com (T.-T.W.); liujingyi9803@163.com (J.-Y.L.); guoruigrace@163.com (R.G.); sl0729an@163.com (J.-D.A.); hxxyhjz@tjnu.edu.cn (J.-Z.H.); liuyuanyuan1973@aliyun.com (Y.-Y.L.); 2 Department of Chemistry and Key Laboratory of Advanced Energy Materials Chemistry, College of Chemistry, Nankai University, Tianjin 300071, China

**Keywords:** metal-organic frameworks, fluorescence, fluorescent probe, antibiotics, amino acid

## Abstract

Metal-organic frameworks (MOFs) have been rapidly developed for their broad applications in many different chemistry and materials fields. In this work, a multi-dentate building block 5-(4-(tetrazol-5-yl)phenyl)-isophthalic acid (H_3_L) containing tetrazole and carbolxylate moieties was employed for the synthesis of a two-dimensional (2D) lanthanide MOF [La(HL)(DMF)_2_(NO_3_)] (DMF = *N*,*N*-dimethylformamide) (**1**) under solvothermal condition. The fluorescent sensing application of **1** was investigated. **1** exhibits high sensitivity recognition for antibiotic nitrofurantoin (K_sv_: 3.0 × 10^3^ M^−1^ and detection limit: 17.0 μM) and amino acid l-tyrosine (K_sv_: 1.4 × 10^4^ M^−1^ and detection limit: 3.6 μM). This work provides a feasible detection platform of 2D MOFs for highly sensitive discrimination of antibiotics and amino acids.

## 1. Introduction

Metal-organic frameworks (MOFs) are coordination compounds with open-framework structure. In the past few decades, MOFs have been widely studied in many fields such as gas storage and separation, adsorption and separation of small chemical species, sensing, catalysis and drug delivery [1,2,3,4,5,6]. By improving the synthesis method, adjusting the proportion of metal salts, ligands and solvents, and changing the pore structures or porosities, the catalytic, gas adsorption and separation or fluorescence performance can be optimized [7,8,9]. MOFs can be used as chemical sensors because the interactions between MOFs and analytes could influence their luminescent properties [10,11,12]. Usually, the chemical sensors could have a “turn on” or “turn off” response to small molecules [13,14,15]. The characteristic of a good sensor is usually summarized as “4S”: sensitivity, selective, stability and speed of response [16,17,18,19,20,21,22].

Since antibiotics were discovered, these molecules have showed applications for disease cure. However, antibiotic pollution has become more and more serious, which is caused by the abuse of antibiotics [23,24,25]. Nitrofurantoin is an antibiotic that is widely used in the prevention and treatment of animal infectious diseases [26,27], but it has been banned in many countries because of its carcinogenicity and mutagenicity. In 2008, the Ministry of Agriculture of China set the maximum limit of nitrofurantoin in aquatic products at 0.5 μg/kg [28].

On the other hand, l-tyrosine is an essential amino acid. The deficiency of l-tyrosine may cause phenylketonuria (PKU) [29,30]. PKU is an inborn metabolic error that prevents the conversion of l-tyrosine, causing damage to the central nervous system [31,32]. In addition, l-tyrosine is associated with dopamine (associated with Parkinson’s disease), norepinephrine and epinephrine synthesis [33].

**Scheme 1:**
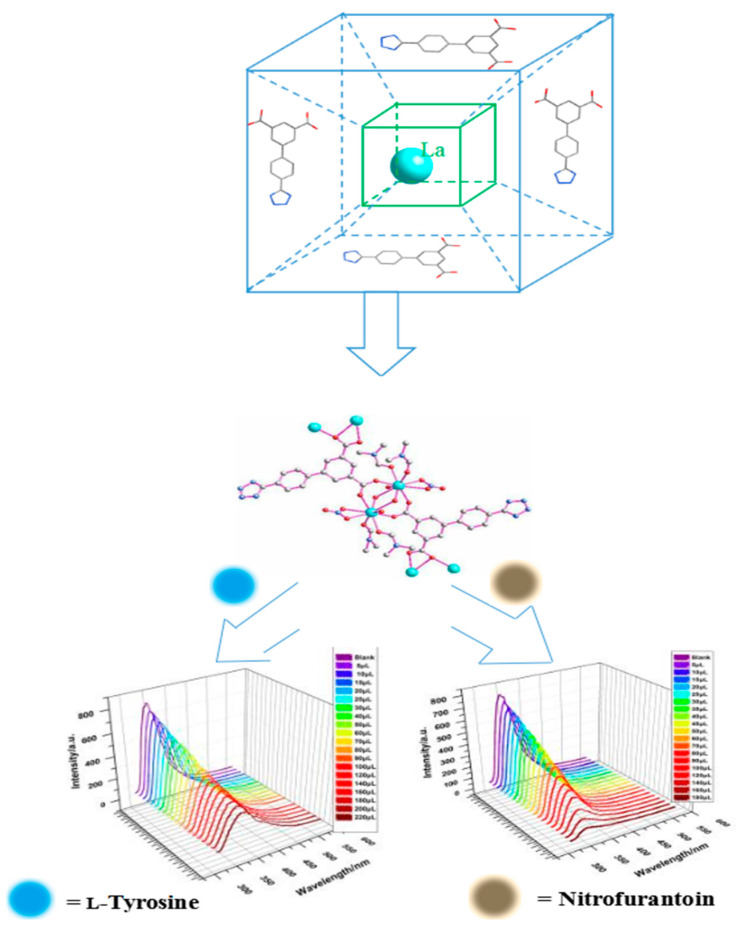
Preparation of the lanthanide metal-organic framework for highly sensitive discrimination of nitrofurantoin and l-tyrosine.

Based on the above consideration, we are interested in developing fluorescent probes for both nitrofurantoin and l-tyrosine [34,35,36]. In this work, 5-(4-(tetrazol-5-yl)phenyl)-isophthalic acid (H_3_L) containing tetrazole and carbolxylate moieties was employed as a bridging ligand. Through the solvothermal method, a lanthanide MOF, [La(HL)(DMF)_2_(NO_3_)] (**1**) was synthesized. We investigated the photo-luminescence sensing performance of **1**, which showed highly sensitive sensing function for both nitrofurantoin (K_sv_: 3.0 × 10^3^ M^−1^ and detection limit: 17.0 μM) and l-tyrosine (K_sv_: 1.4 × 10^4^ M^−1^ and detection limit: 3.6 μM) with high quenching efficiency and low detection limit (Scheme 1).

## 2. Results and Discussion

### 2.1. Structural Description of ***1***

**Figure 1:**
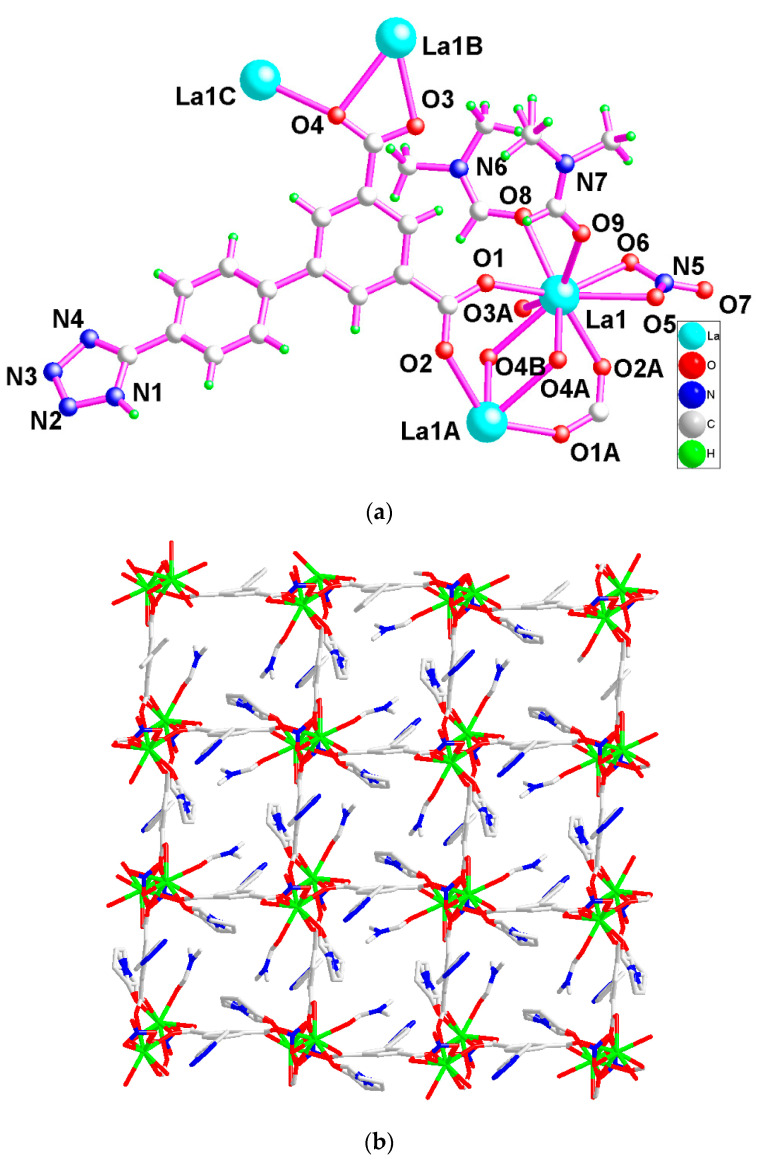
(**a**) The fundamental structure of **1** (symmetry codes: A 0.5 − x, 1.5 − y, 1 − z; B x,1 − y, 0.5 + z; C x,1 − y, 0.5 + z.); (**b**) two-dimensional framework of **1**.

**1** belongs to monoclinic space group *C2/c.* As shown in Figure 1a, the asymmetric unit of **1** consists of one La^III^ center (La1), one de-protonated HL^−^, two mono-coordinated DMF and one bi-coordinated NO_3_^−^. The HL^−^ ligand serves as a μ_4_-bridge to link four La centers by a μ_4_-η^1^:η^1^:η^1^:η^2^ coordination mode through four carboxylate oxygen atoms (O1, O2, O3 and O4). Two DMF molecules are mono-coordinated to La1 through the terminal oxygen atoms (O8 and O9). Besides, two oxygen atoms (O5 and O6) from the nitrate ion are coordinated to La1 forming a bidentate chelating coordination mode [37,38]. The bond lengths of La-O are in the range of 2.443(2)–2.741(2) Å. The angle range of O-La-O is 72.49(7)–151.40(8)°. All the bond lengths and angles fell into the normal range.

Figure 1b shows that two neighboring lanthanide atoms (La1A and La1B) are connected by the carboxylate oxygen atoms (O3 and O4) of HL^−^ forming a binuclear building block. The intermetallic distance of La1A and La1B is 4.1258(4) Å. These binuclear building blocks are further linked through HL^−^ to form a two-dimensional network. It is noted that the tetrazole group of HL^−^ is not coordinated and protruded out of the two-dimensional network. There are hydrogen bonding interactions between the two-dimensional networks: N(1)–H(1)···O(5), 2.9651(1) Å and C(3)–H(3)···O(7), 3.3256(1) Å. The hydrogen-bonding interactions further assemble the two-dimensional networks into a three-dimensional supramolecular structure [39].

### 2.2. PXRD, FT-IR and SEM Characterizations of ***1***

**Figure 2:**
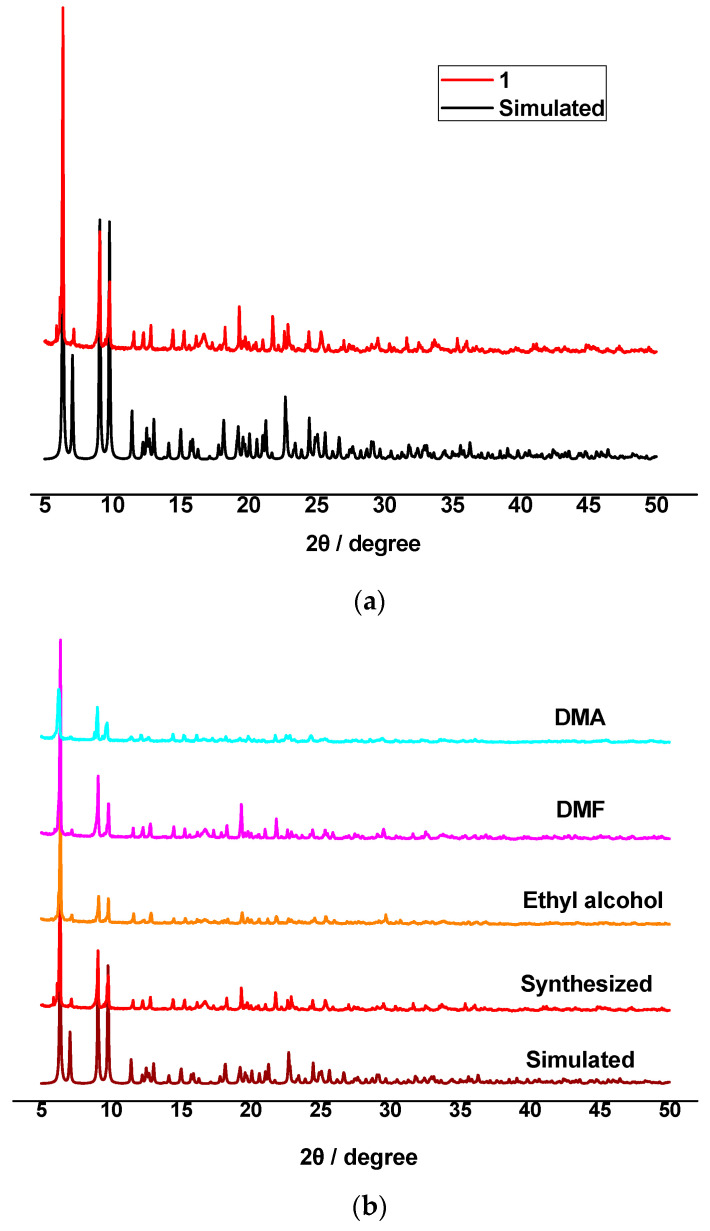
(**a**) PXRD patterns of **1**; (**b**) PXRD patterns of **1** soaked in different solutions for 24 h.

The powder X-ray diffraction (PXRD) pattern of **1** is shown in Figure 2a. The experimental peaks are consistent with the theoretical one obtained by single-crystal X-ray data [40,41,42]. We also investigated the stability of **1** in different solvents such as ethanol, DMF and DMA. After 24 h soaking **1** in these solvents, the experimental PXRD patterns of **1** were also consistent with theoretical pattern, indicating that **1** was stable in these solvents (Figure 2b). The slight variation of diffraction intensity may be related to the different crystal orientation or solvent effects [43,44].

FT-IR spectrum of **1** in the range of 4000–400 cm^−1^ was measured (Figure S1, Supplementary Materials). The strong and wide peak close to 3412 cm^−1^ can be ascribed to the presence of N–H stretching vibration. The strong peaks of carboxyl groups appear in the region of 1648–1584 cm^−1^ (antisymmetric stretching vibrations) and 1384–1420 cm^−1^ (symmetric stretching vibrations) [45,46,47]. No FT-IR peaks around 1700 cm^−1^ also demonstrated complete deprotonation of carboxyl groups [48,49,50,51,52]. The peaks located at 1499 cm^−1^ are from the tetrazole group. The band located at 1296 cm^−1^ can be ascribed to the vibration of C–N [53,54,55]. The vibration bands from 779 cm^−1^ to 719 cm^−1^ are from the aromatic benzene rings.

**Figure 3:**
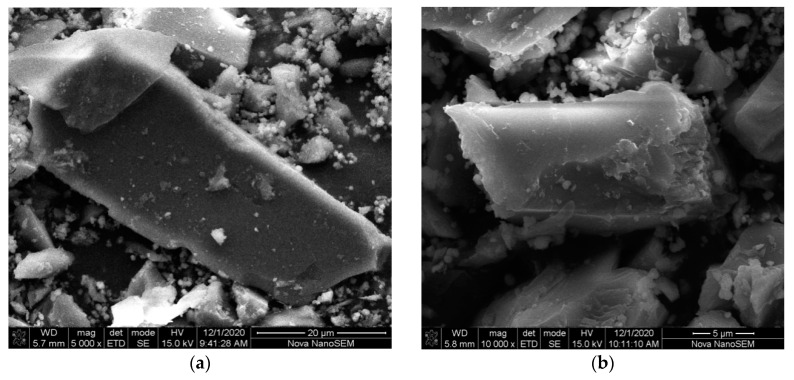
(**a**) The SEM image of **1** at a scale of 20 μm; (**b**) The SEM image of **1** at a scale of 5 μm.

The morphology of **1** was also investigated by the scanning electron microscope (SEM) [56,57,58,59,60,61,62]. As shown in Figure 3a,b, **1** has a blocky morphology with a length of 54.94 μm and a width of 21.38 μm [63,64,65,66,67], which is different to that of H_3_L (Figure S2a,b, Supplementary Materials) under the same scale.

### 2.3. Photo-Luminescent Properties of ***1***

It is well known that luminescence can be divided into two basic modes according to the spin state of electrons during radiation relaxation: fluorescence and phosphorescence [68,69,70]. The origin of lanthanide MOFs can be mainly ascribed to four kinds of mechanisms listed below: (1) the luminescence based on the organic ligand, (2) the luminescence based on metal center, (3) the luminescence based on the charge transfer, and (4) the luminescence based on guest emission. Lanthanide metal ions contain 4f electrons shielding by 5s^2^5p^6^ orbits, which is hardly perturbed by its chemical surroundings, which enable lanthanide ions to have good optical performance [71,72,73].

UV-Vis spectra of H_3_L and **1** were measured at room temperature (Figure S3, Supplementary Materials). The maximum absorption peak positions of H_3_L and **1** were different, which can be ascribed to the coordination between H_3_L and the metal center. Solid-state fluorescence spectra of H_3_L and **1** were also measured, as shown in Figure S4, Supplementary Materials. The peak value of **1** appeared at the same wavelength of H_3_L excited at 300 nm, indicating that the fluorescence emission of **1** is from the ligand [74,75,76].

**Figure 4:**
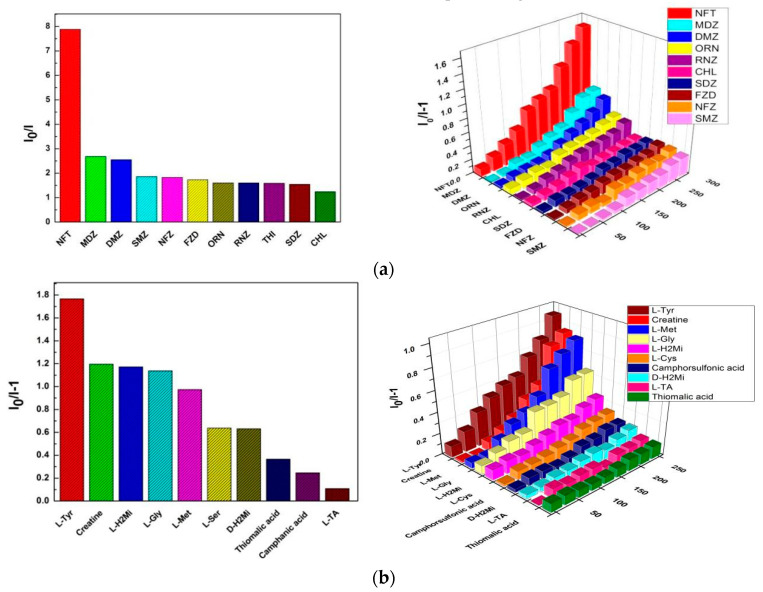
(**a**) Photo-luminescent intensities at 353 nm for **1** in the presence of different antibiotics; (**b**) photo-luminescent intensities at 353 nm for **1** in the presence of different amino acids.

In order to explore different photo-luminescence responses to antibiotics [77,78], the powder of **1** was evenly distributed in ethanol solution with a concentration of 0.1 mg/mL by the ultrasonic method at room temperature (ultrasonic power: 100 W; ultrasonic time: 20 min). The antibiotics used are listed below: nitrofurantoin (NFT), ronidazole (RNZ), furazolidone (FZD), nitrofurazone (NFZ), dimetridazole (DMZ), ornidazole (ORN), thiamphenicol (THI), metronidazole (MDZ), chloramphenicol (CHL), sulfamethazine (SMZ) and sulfadiazine (SDZ). In the fluorescence detection experiments, different antibiotics with the concentration of 0.001 mol/L were added to the 3 mL suspended solutions containing **1**, drop by drop. The fluorescence quenching results are shown in Figure 4a. The results show that the quenching efficiency is different, among which NFT shows the most obvious quenching result.

On the other hand, diverse amino acids were added into the suspensions of **1** to study the photo-luminescence response of **1**. Different amino acids, including l-tyrosine (l-Tyr), creatine, l-malic acid(l-H_2_MI), d-malic acid (d-H_2_MI), l-methionine (l-Met), l-glycine(l-Gly), l-cysteine (l-Cys), camphorsulfonic acid, l-tartaric acid(l-TA) and thiomalic acid were studied. As shown in Figure 4b, l-Tyr showed the most obvious fluorescence quenching result. The fluorescence emission intensities of **1** are highly depended on the addition of l-Tyr, indicating that **1** could be used as the fluorescence probe of l-Tyr.

**Figure 5:**
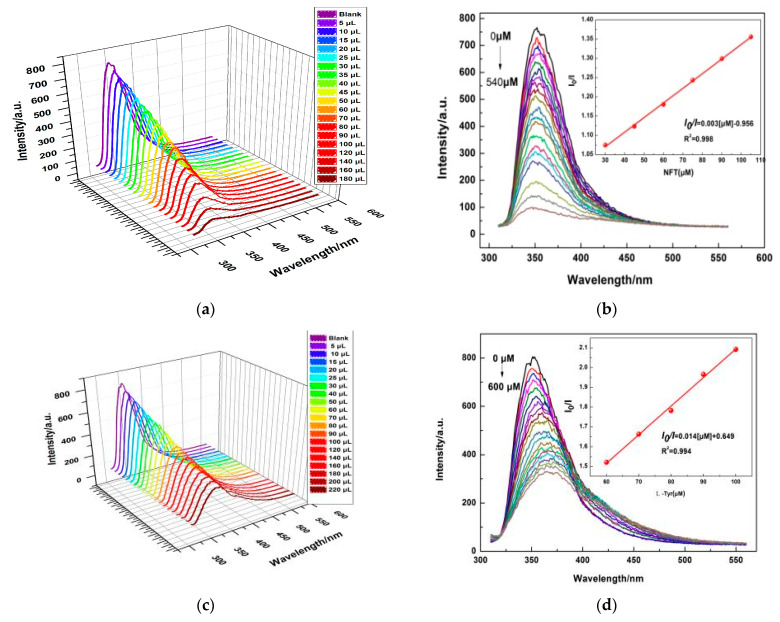
(**a**) The luminescence spectra of **1** with different concentrations of NFT; (**b**) The emission spectra by adding different concentration NFT buffer excited at 300 nm; (**c**) The luminescence spectra of **1** under different concentrations of l-Tyr; (**d**) The emission spectra by adding different concentration l-Tyr buffer excited at 300 nm.

The Stern–Volmer (SV) curves for NFT and l-Tyr were plotted, respectively [79]. The SV curves of NFT and l-Tyr were approximately linear in the concentration range of 0–0.54 and 0–0.6 mM, respectively. *I*_0_/*I* = K_sv_[A] + 1 was used to determine the value of K_sv_, in which *I* and *I*_0_ represent the emission intensities with or without NFT or l-Tyr, [A] is the concentration of NFT or l-Tyr, and K_sv_ is the quenching coefficient. The detection limit of NFT/l-Tyr is 17.0/3.6 μM and K_sv_ is 3.0 × 10^3^/1.4 × 10^4^ M^−1^ (Figure 5). The PXRD patterns of **1** immersed in the ethanol solution of NFT or l-Tyr for 24 h are in good agreement with the simulated PXRD of **1**, indicating the structure of **1** did not collapse in the detection process (Figure S5, Supplementary Materials). Therefore, **1** is a good candidate as a fluorescence probe for NFT or l-Tyr.

**Figure 6:**
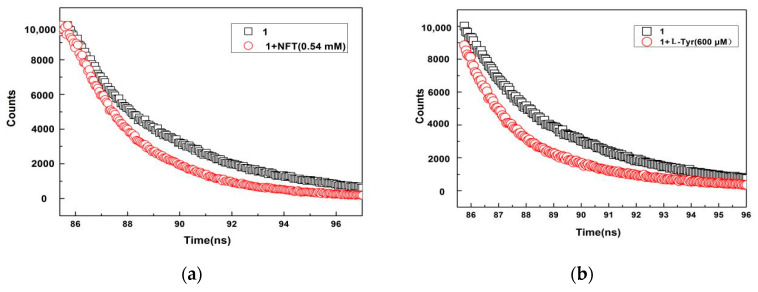
(**a**) The decay curve of **1** with the addition of NFT; (**b**) The decay curve of **1** with the addition of l-Tyr.

Fluorescence lifetime is an important parameter for judging the mechanism of fluorescence quenching [80]. We investigated the fluorescence lifetimes for the suspensions of **1** with the addition of NFT or l-Tyr at room temperature, as shown in Figure 6 and Table 1. When NFT or l-Tyr was added into the suspensions of **1,** the fluorescence lifetimes change from 4.44 ns to 4.77 ns or 2.82 ns, indicating the existence of a dynamic fluorescent quenching mechanism. The results also suggested that NFT and l-Tyr may interact with **1** through weak interactions such as hydrogen bonding interactions. Considering that the photo-luminescent origin of **1** is from ligand-based luminescent emission and the 2D coordination framework of **1** have uncoordinated tetrazole moieties of HL^−^, these uncoordinated tetrazole moieties could have hydrogen bonding interactions with the analytic species, which can reduce the photo-luminescent emission of **1** and cause the dynamic quenching phenomenon [81].

**Figure 7:**
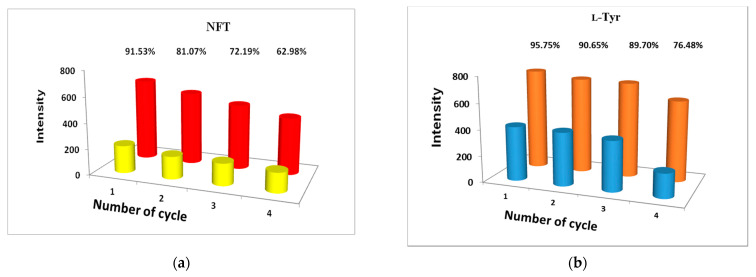
Cycling performance of luminescence intensities at 300 nm of **1** for detecting NFT (**a**) and l-Tyr (**b**).

We also investigated the cycling performance of **1** (Figure 7). After four cycles, the efficiency of NFT or l-Tyr was 62.98 or 76.48%, indicating medium cycling performance.

## 3. Materials and Methods

### 3.1. General Remarks

H_3_L was purchased from Jinan Henghua Technology Co., Ltd., Shan Dong, China. All other chemicals were purchased commercially and applied directly. Perkin-Elmer 240 element analyzer (PerkinElmer, Dublin, Ireland) is used for microanalysis of C, H and N elements. The ultraviolet-visible spectrophotometer (model UV-2600) manufactured by Shimadzu Company (Shimadzu, Kyoto, Japan), was used to measure the absorption spectra at room temperature, with the test wavelength ranging from 200–800 nm. Powder X-ray diffraction analysis was characterized by Rigaku D/Max-2500 (Rigaku, Tokyo, Japan) X-ray diffractometer equipped with Cu-Kα radiation at a wavelength of 0.154 nm. Photo-luminescence lifetimes and solid-state fluorescence spectra were measured by FS5 fluorescence spectrometer (Edinburgh Instruments, Edinburgh, UK). The RF-5301 fluorescence spectrophotometer (Shimadzu, Kyoto, Japan) was used to carry out the photo-luminescence sensing experiment, equipped with a plotter unit and 1 cm × 1 cm quartz battery in phosphorescent mode.

### 3.2. Preparation of [La(HL)(DMF)_2_(NO_3_)] *(**1**)*

**Scheme 2:**
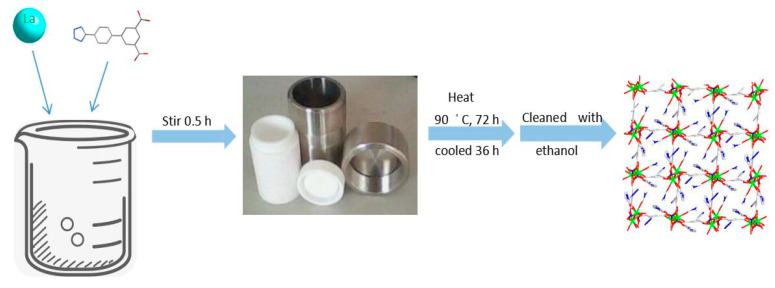
Synthesis route for **1**.

La(NO_3_)_2_·6H_2_O (129.9 mg, 0.3 mmol), H_3_L (31.0 mg, 0.1 mmol), ethanol (3 mL) and DMF (1 mL) were added to a beaker and stirred for 0.5 h, and then the mixture was transferred to a steel high-pressure reaction kettle which is heated to 90 °C for 72 h, and cooled to the ambient temperature within 36 h. The resulting pale yellow powder was cleaned with ethanol several times (Scheme 2). At room temperature, the powder of **1** was evenly distributed in ethanol solution with a concentration of 0.1 mg/mL by the ultrasonic method, which was used for further fluorescent measurement. Yield: 36% based on H_3_L. Elemental analysis calculations (%) for C_21_H_22_LaN_7_O_9_: C 38.49, H 3.38, N 14.96; Found: C 38.57, H 3.49, N 15.13. FT-IR data (cm^−1^, KBr): 3412(w), 1649(m), 1621(m), 1584(w), 1500(w), 1449(w), 1421(w), 1384(s), 1296(w), 1112(w), 779(w), 719(w), 674(w). The prepared sample needs to be pretreated before FT-IR and other characterization.

### 3.3. X-ray Crystallography

The Bruker SMART 1000 CCD diffractometer (Bruker, AVANCE, Billerica, MA, USA) was used to measure the diffraction data of a single crystal of **1**, equipped with graphite monochromatic Mo-Kα aperture radiation (λ = 0.71073 Å). The ω-φ scanning strategy was applied with Lorentz polarization and empirical absorption correction. By utilizing the SHELXS97 program, the structure of F^2^ was improved by utilizing a full matrix least squares calculation. The anisotropic temperature factor was assigned to all atoms except hydrogen atoms, and the isotropic temperature factor was arbitrarily selected to be 1.2 times that of the parent [82,83]. The R(F), WR(F^2^) and goodness of fit protocol factors, details of data collection and analysis are shown in Table 2. The selected bond lengths, angle hydrogen bonds [Å] and angles [°] are given in Table S1, Supplementary Materials. CCDC-1873742 represents the crystal data for this work. The data is available free of charge through the Cambridge Crystal Data Center.

## 4. Conclusions

In summary, the preparation, structural characterization and photo-luminescent sensing performance of a 2D lanthanide metal-organic framework was reported. It can be utilized to detect trace nitrofurantoin (K_sv_: 3.0 × 10^3^ M^−1^ and detection limit: 17.0 μM) and l-tyrosine (K_sv_: 1.4 × 10^4^ M^−1^ and detection limit: 3.6 μM) with high sensitivity and good recyclability. This work not only enriches the research of lanthanide MOFs, but also provides a theoretical basis for 2D MOF-based chemical sensors.

## Figures and Tables

**Table 1:** The lifetimes of **1** with or without the addition of NFT and l-Tyr.

**Caption**	**T_1_/ns**	**T_2_/ns**	**B_1_/%**	**B_2_/%**	**Combined Life Rating/ns**
1	3.487773	6.584965	69.38	30.62	4.436
1 + NFT	1.997302	3.409927	41.78	58.22	2.820
1 + l-Tyr	2.528950	10.37314	71.39	28.61	4.773

**Table 2:** Crystal Data and Structure Refinement Information for **1**.

1	
Formula	C_21_H_22_LaN_7_O_9_
*M* (g·mol^−1^)	655.36
Crystal system	Monoclinic
Space group	*C2/c*
Temperature	133.32(16)
*a* (Å)	28.2179(10)
*b* (Å)	14.0169(5)
*c* (Å)	14.3437(5)
α (°)	90
β (°)	99.720(4)
γ (°)	90
*V* (Å^3^)	5591.9(3)
*Z*	8
*F* (000)	2608
*ρ*_calc_ (Mg·m^−3^)	1.557
*μ* (mm^−1^)	1.586
Data/restraints/parameters	4916/36/362
GOF on *F*^2^	1.069
*R*_1_^a^ (I ≥ 2σ(*I*))	0.0273
w*R*_2_^b^ (all data)	0.0643

^a^*R*_1_ = Σ||F_o_| − |F_c_||/|F_o_|, ^b^ w*R*_2_ = [Σw(F_o_^2^ − F_c_^2^)^2^/Σw(F_o_^2^)^2^]^1/2^.

## Data Availability

The data presented in this study are available with the authors.

## Supplementary Materials

Table S1: Selected bond lengths (Å) and angles (°), Figure S1: FT-IR spectrum of **1**, Figure S2: (**a**) The SEM images of H_2_L showed that the powder is needle-like with the length between 0.54–5.99 m at a scale of 5 μm (**b**) The SEM images of H_2_L showed that the powder is acicular on a 2 μm scale., Figure S3: UV-Vis spectra of H_3_L and **1****,** Figure S4: Solid-state fluorescence of H_3_L and **1**, Figure S5: powder X-ray patterns for **1**, **1** + NFT and **1** + l-Tyr.

## Sample Availability

Sample of the compound **1** is available from the authors.
