# Supplementary material for: Solvothermal Preparation of a Lanthanide Metal-Organic Framework for Highly Sensitive Discrimination of Nitrofurantoin and l-Tyrosine
Source: Molecules. 2021 Jun 16;26(12):3673. doi: 10.3390/molecules26123673 (PMC8233945; doi:10.3390/molecules26123673)
Supplement: Supplementary file 1 [file molecules-26-03673-s001.zip › molecules-1241121-supplementary.pdf]

molecules

MDPI

Supplementary Materials

Solvothermal Preparation of a Lanthanide Metal-Organic Framework for Highly Sensitive Discrimination of Nitrofurantoin and L-Tyrosine

Tian-Tian Wang [1], Jing-Yi Liu [1], Rui Guo [1], Jun-Dan An [1], Jian-Zhong Huo [1], Yuan-Yuan Liu [1], Wei Shi [2,*] and Bin Ding [1,*]

[1] Key Laboratory of Inorganic-Organic Hybrid Functional Material Chemistry, College of Chemistry, Tianjin Normal University, 393 Binshui West Road, Tianjin 300387, China; tiantianwang0912@163.com (T.-T.W.); liujingyi9803@163.com (J.-Y.L.); guoruigrace@163.com (R. G.); sl0729an@163.com (J.-D.A.); hxxy-hjz@tjnu.edu.cn (J.-Z.H.); liuyuanyuan1973@aliyun.com (Y.-Y.L.)

[2] Department of Chemistry and Key Laboratory of Advanced Energy Materials Chemistry, College of Chemistry, Nankai University, Tianjin 300071, China

* Correspondence: hxxydb@tjnu.edu.cn (B.D.); shiwei@nankai.edu.cn (W.S.)

Table S1. Selected Bond Lengths (Å) and Angles (°) for **1.**

		1			
La(1)- O(4)	2.741(2)	La(1)- O(4)	2.460(2)	La(1)- O(6)	2.592(2)
La(1)- O(2)	2.5018(19)	La(1)- O(5)	2.681(2)	La(1)- O(3)	2.534(2)
La(1)- O(1)	2.443(2)	La(1)- O(8)	2.521(2)	La(1)- O(9))	2.491(2)
O(4)-La(1)- O(5)	84.58(7)	O(4)-La(1)- O(2)	72.49(7)	O(4)-La(1)- O(3)	120.83(7)
O(4)-La(1)- O(9)	91.63(7)	O(4)-La(1)- O(6)	130.55(7)	O(4)-La(1)- O(8)	146.57(8)
O(2)-La(1)- O(5)	64.75(7)	O(2)-La(1)- O(3)	73.99(7)	O(2)-La(1)- O(4)	73.72(7)
O(2)-La(1)- O(6)	73.05(7)	O(5)-La(1)- O(4)	137.63(6)	O(2)-La(1)- O(8)	138.51(7)
O(1)-La(1)- O(4)	73.56(7)	O(1)-La(1)- O(2)	135.00(7)	O(1)-La(1)- O(5)	138.88(7)
O(1)-La(1)- O(3)	99.74(7)	O(1)-La(1)- O(8)	73.63(8)	O(1)-La(1)- O(9)	75.19(7)
O(8)-La(1)- O(4)	99.21(7)	O(8)-La(1)- O(5)	117.10(8)	O(8)-La(1)- O(3)	71.31(7)
O(9)-La(1)- O(4)	144.64(7)	O(9)-La(1)- O(2)	133.93(7)	O(9)-La(1)- O(5)	71.01(7)
O(6)-La(1)- O(5)	48.68(7)	O(9)-La(1)- O(8)	73.91(8)	O(6)-La(1)- O(4)	126.06(7)

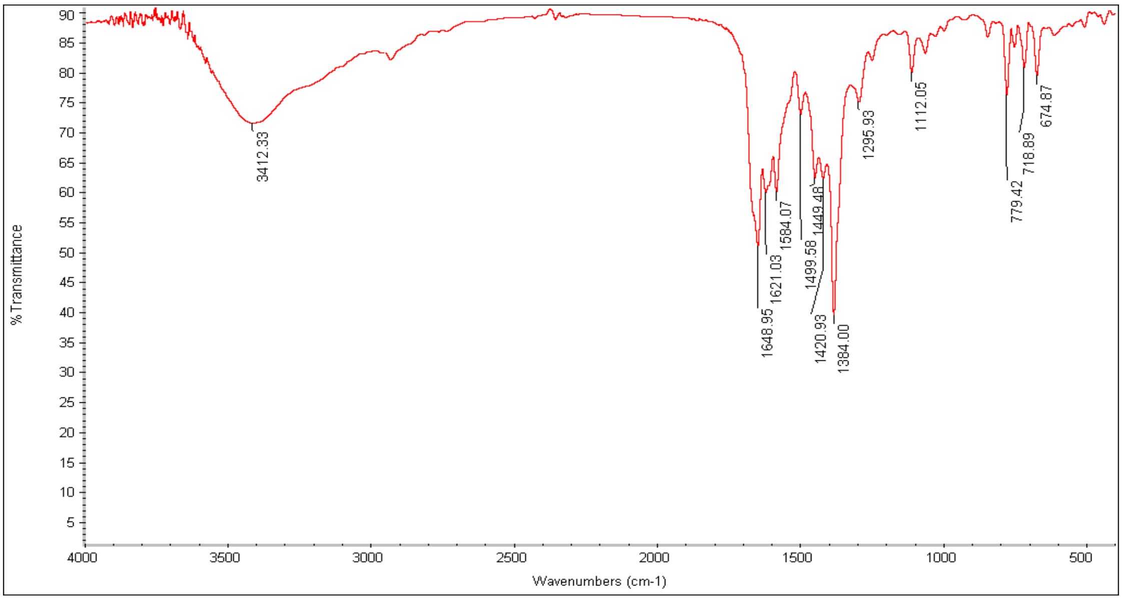

Figure S1. FT-IR spectra of coordination material **1**.

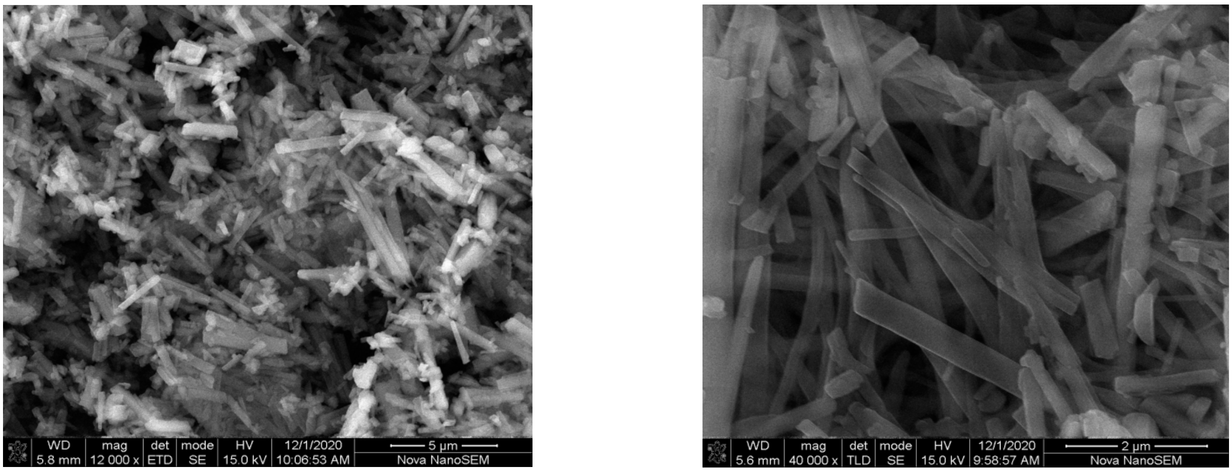

(**a**) (**b**)

Figure S2. (**a**) The SEM images of H_2L showed that the powder is needle-like with the length between 0.54–5.99 m at a scale of 5 μm (**b**) The SEM images of H_2L showed that the powder is acicular on a 2 μm scale.

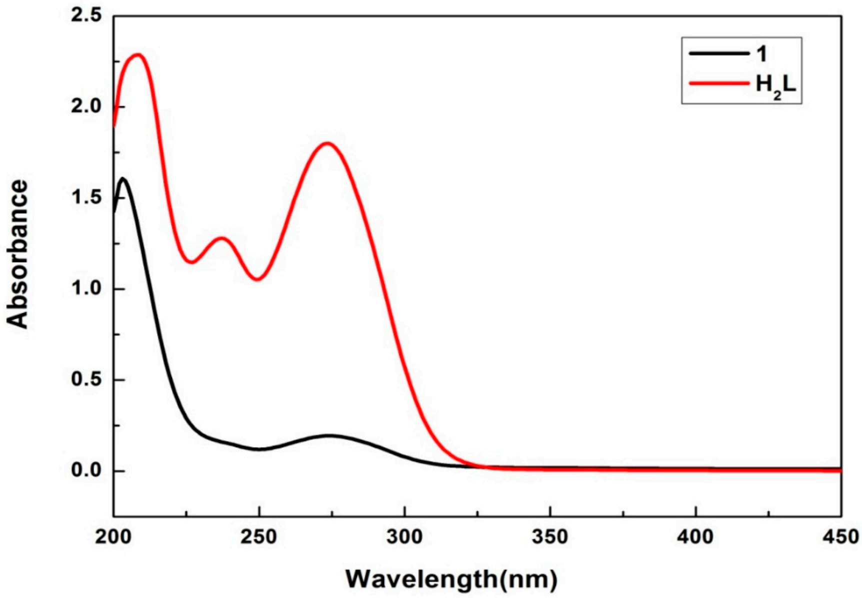

Figure S3. UV-Vis spectra of free H_2L and **1**.

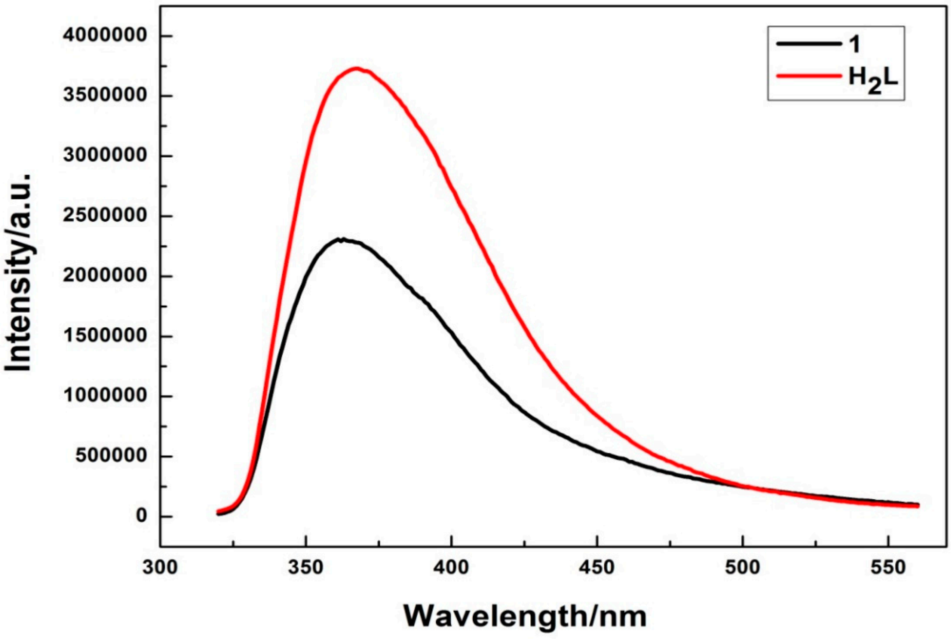

Figure S4. Solid-state fluorescence of free H_3L and **1**.

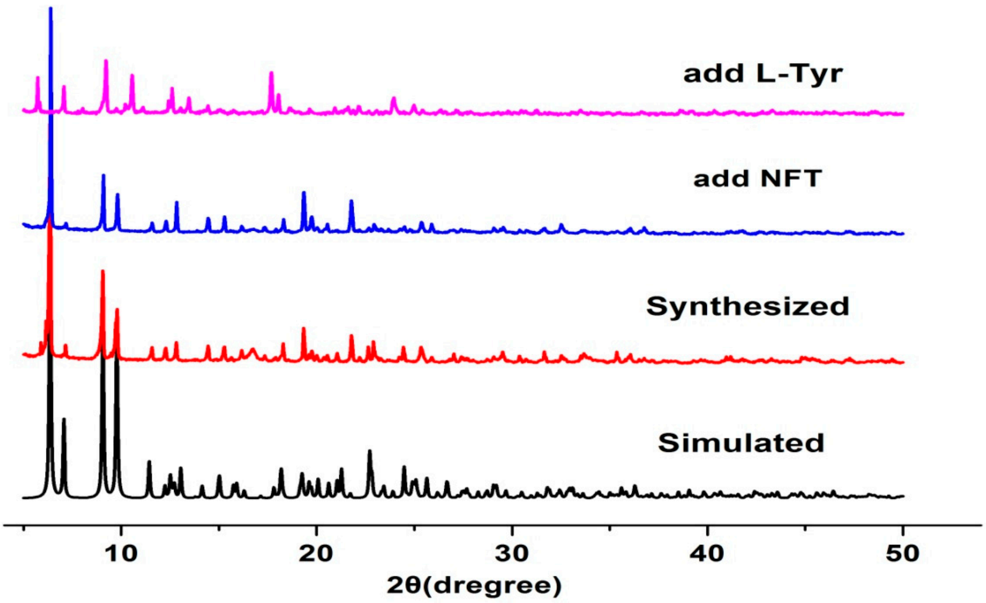

Figure S5. Powder X-Ray patterns for **1**, **1** + NFT and **1** + L-Tyr.
